# Identification of Volatile Sulfur Compounds Produced by *Schizophyllum commune*

**DOI:** 10.3390/jof7060465

**Published:** 2021-06-08

**Authors:** Takahito Toyotome, Masahiko Takino, Masahiro Takaya, Maki Yahiro, Katsuhiko Kamei

**Affiliations:** 1 Department of Veterinary Medicine, Obihiro University of Agriculture and Veterinary Medicine, 2-1 Inada-cho Nishi, Obihiro, Hokkaido 080-8555, Japan; 2 Diagnostic Center for Animal Health and Food Safety, Obihiro University of Agriculture and Veterinary Medicine, Inada-cho, Obihiro, Hokkaido 080-8555, Japan; 3 Medical Mycology Research Center, Chiba University, 1-8-1 Inohana, Chuo-ku, Chiba, Chiba 260-8673, Japan; myahiro@office.ac.jp (M.Y.); k.kamei@faculty.chiba-u.jp (K.K.); 4 Japan Application Center, Life Sciences and Chemical Analysis, Agilent Technologies Japan, Ltd., 9-1 Takakura-cho, Hachioji, Tokyo 192-8510, Japan; masahiko_takino@agilent.com; 5 The Tokachi Foundation, Obihiro, Hokkaido 080-2462, Japan; takaya@food-tokachi.jp; 6 Graduate School of Animal and Veterinary Sciences and Agriculture, Obihiro University of Agriculture and Veterinary Medicine, Inada-cho, Obihiro, Hokkaido 080-8555, Japan

**Keywords:** allergic bronchopulmonary mycosis, microbial volatile organic compounds, *Schizophyllum commune*, volatile sulfur compounds

## Abstract

*Schizophyllum commune* is a causative agent of allergic bronchopulmonary mycosis, allergic fungal rhinosinusitis, and basidiomycosis. Diagnosis of these diseases remains difficult because no commercially available tool exists to identify the pathogen. Unique volatile organic compounds produced by a pathogen might be useful for non-invasive diagnosis. Here, we explored microbial volatile organic compounds produced by *S. commune*. Volatile sulfur compounds, dimethyl disulfide (48 of 49 strains) and methyl ethyl disulfide (49 of 49 strains), diethyl disulfide (34 of 49 strains), dimethyl trisulfide (40 of 49 strains), and dimethyl tetrasulfide (32 of 49 strains) were detected from headspace air in *S. commune* cultured vials. Every *S. commune* strain produced at least one volatile sulfur compound analyzed in this study. Those volatile sulfur compounds were not detected from the cultures of *Aspergillus* spp. (*A. fumigatus*, *A. flavus*, *A. niger*, and *A. terreus*), which are other major causative agents of allergic bronchopulmonary mycosis. The last, we examined H_2_S detection using lead acetate paper. Headspace air from *S. commune* rapidly turned the lead acetate paper black. These results suggest that those volatile sulfur compounds are potent targets for the diagnosis of *S. commune* and infectious diseases.

## 1. Introduction

A basidiomycetous fungus *Schizophyllum commune* is a common mushroom that is found frequently in the environment, especially on withered trees. The fungus is also known as a causative agent of allergic bronchopulmonary mycosis (ABPM), allergic fungal rhinosinusitis, and basidiomycosis [1,2,3]. In contrast to fungal allergy, those diseases involve the colonization and growth of the causative agent in the host. A nationwide survey revealed that sputum culture among 6% of the patients with possible ABPA-central bronchiectasis was *S. commune* positive [4], although *Aspergillus* spp. are the major causative agents of ABPA. Chowdhary reviewed 218 reported cases of basidiomycosis including allergic and invasive mycoses, which showed that the most common causative agent among basidiomycetes was *S. commune* (52.3%) [2].

*S. commune* is usually identified by culture methods. However, morphological identification of *S. commune* is difficult, and colonies may be considered simply contaminated or misidentified as other causative agents because the colony shows non-specific, white, and fluffy characteristics. Further, it seems to be rare to successfully culture *S. commune* from clinical specimens. We previously identified a *S. commune* antigen *Sch* c1 [5]. Although the increased titer against *Sch* c1 was auxiliary used for the diagnosis of *S. commune* infection, serum samples are required for the method [6]. Further options, especially non-invasive and rapid options, are being awaited for for the diagnosis of diseases by *S. commune*.

The culture emits an unpleasant odor derived from volatile organic compounds (VOCs) [2], suggesting that unique VOCs are promising biomarkers for use in the diagnosis of *S. commune* infection. VOCs produced by *Aspergillus* spp., the major causative agent of ABPM, have been investigated [7,8,9]. Syhre et al. reported 2-pentylfuran as a VOC, suggesting it as a biomarker candidate of *Aspergillus* infection [7,10,11]. Other compounds such as α/β-trans-bergamotene, trans-geranylacetone [8], 2,3-butanedione, and 3-hydroxy-2-butanone [9] were also reported as VOCs produced by *A. fumigatus*. Although several VOCs produced from *S. commune* were identified [12,13], few reports have been published. Schalchli showed ethanol and β-bisabolol as the major VOCs from *S. commune* [13]. Freihorst et al. reported that methyl 2-methylbutanoate, ethyl 2-methylbutanoate, and methyl 2-methylpropanoate were detected from monokaryotic mycelia of *S. commune* and some VOCs, such as *S*-methyl thioacetate and 3-methyl-1-butanol, were detected specifically from dikaryon [12].

As explained in earlier reports [7], VOCs might be potential biomarkers of the infection. For this study, we explored *S. commune* VOCs that might be candidates as biomarkers of infectious diseases by *S. commune*.

## 2. Materials and Methods

### 2.1. Strains and Reagents

*S. commune* strains and *Aspergillus* strains used for this study are shown in Table S1. All strains were provided by the Medical Mycology Research Center, Chiba University. The strains were cultured routinely on potato dextrose agar (PDA; Becton, Dickinson and Co., Franklin Lakes, NJ, USA) slants with air-permeable silicone rubber plugs at 35 °C in an incubator. These strains were handled in a biosafety cabinet Class II type A2 (PHC Co., Tokyo, Japan). Dimethyl disulfide and dimethyl trisulfide were purchased from Fujifilm Wako Pure Chemical Co. (Osaka, Japan). Diethyl disulfide was purchased from Tokyo Chemical Industry Co., Ltd. (Tokyo, Japan).

### 2.2. Solid-Phase Microextraction—Gas Chromatography/Mass Spectrometry

Each strain was inoculated on PDA in a 2 mL vial and cultured under the atmospheric, aerobic conditions. The vial was capped with a crimp cap with a septum to seal securely and was incubated at 35 °C for 29 days. The headspace VOCs of vials were concentrated at 80 °C for 30 min on DVB/Carboxen/PDMS solid-phase microextraction (SPME) fiber (Merck KGaA, Darmstadt, Germany) using MPS2 (Gerstel GmbH & Co. KG, Mülheim an der Ruhr, Germany). Desorption from fiber and gas chromatography (GC) injection was performed at 250 °C for 3 min using a splitless injection method. An Agilent 7890 GC system and a mass spectrometer (MS, Agilent 5975; Agilent Technologies Inc., Santa Clara, CA, USA) were used for analysis. A column DB-5 ms (30 m × 0.25 mm × 0.25 µm) was used for GC analysis. Helium (1.2 mL/min, constant flow) was used as a carrier gas. The oven program was started at 40 °C for 3 min and was then raised to 280 °C at a rate of 10 °C/min. Finally, the temperature 280 °C was reached and held for 5 min. The determination was performed using scan mode in the range of 29–550 Da using an electron ionization (EI) source at 280 °C.

For detection of volatile sulfide compounds at 14 days after culturing *S. commune*, the analysis was performed with slight modifications and different GC–MS. The headspace VOCs of vials were concentrated at 80 °C for 30 min on DVB/carboxen/PDMS SPME fiber (Merck KGaA, Germany). Desorption from fiber and GC injection was performed at 250 °C for 3 min using a splitless injection method. A GC–MS system (GCMS-QP2010 system; Shimadzu, Kyoto, Japan) was used for the analysis. A column Inert Cap1 (30 m × 0.25 mm × 1.5 µm) was used for the GC analysis. Helium (1.2 mL/min, constant flow) was used as a carrier gas. The oven program was started at 40 °C for 3 min and was then raised to 280 °C at a rate of 10 °C/min. Finally, the temperature 280 °C was reached and held for 5 min. The determination was performed using scan mode in the range of 29–550 Da using an electron ionization (EI) source at 260 °C.

### 2.3. H_2_S Detection Using Lead Acetate Paper

PDA was used for culturing fungi in the assay. A piece of lead acetate paper to detect H_2_S as little as 5 ppm in the atmosphere (Merck Millipore, Burlington, MA, USA) was taped to the back of the agar plate lid. A scrape of fungi was inoculated on the center of PDA and was cultured in a gas-barrier pouch (Mitsubishi Gas Chemical Co. Inc., Tokyo, Japan) at 35 °C for four days. The pouch contained atmosphere at the start of culturing, and while culturing was performed under the gas-barrier condition the O_2_ level decreased gradually. The paper was observed three and four days after inoculation. The whiteness of lead acetate paper in RGB images taken using a digital camera (TG-5; Olympus Corp., Tokyo, Japan) was determined using the mean gray value measurement function of ImageJ 1.53a [14]. The values were calculated by converting each pixel to grayscale using the formula gray = 0.299red + 0.587green + 0.114blue in ImageJ.

## 3. Results

### 3.1. Volatile Sulfur Compounds Are the Major VOCs of S. commune

**Figure 1:**
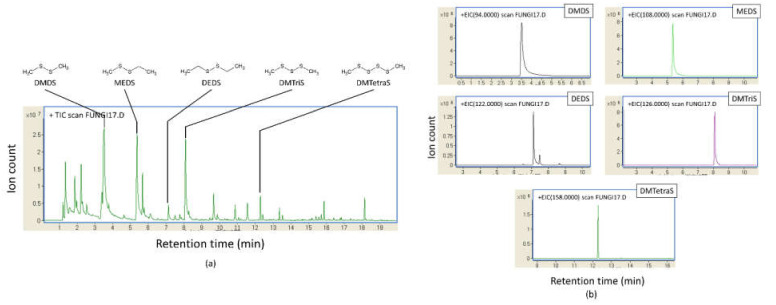
Major polysulfides produced by *S. commune* IFM 59347, a representative strain in which five polysulfides were detected: dimethyl disulfide (DMDS), methyl ethyl disulfide (MEDS), diethyl disulfide (DEDS), dimethyl trisulfide (DMTriS), and dimethyl tetrasulfide (DMTetraS). They are shown in the total ion chromatogram (**a**) and each extracted-ion chromatogram (**b**).

**Figure 2:**
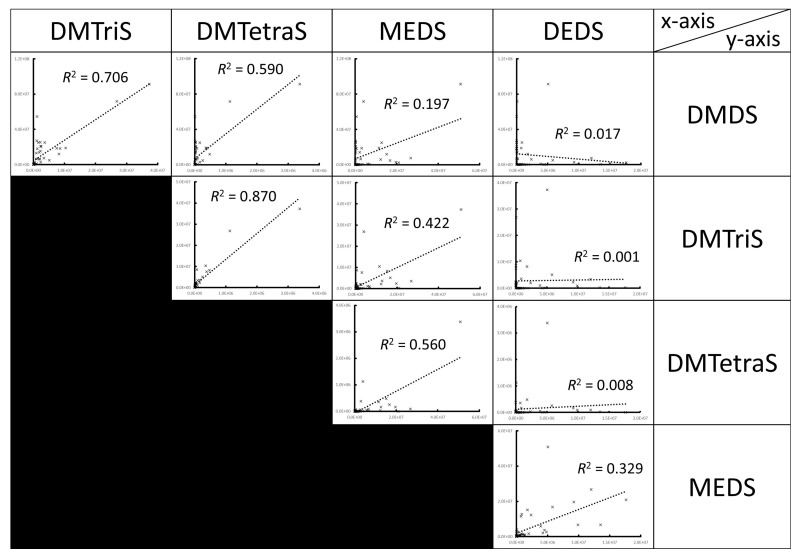
Plots of peak areas of each polysulfide and their respective coefficients of determination. The *x*-axis and *y*-axis in each plot are shown respectively in the top row and the right column.

**Figure 3:**
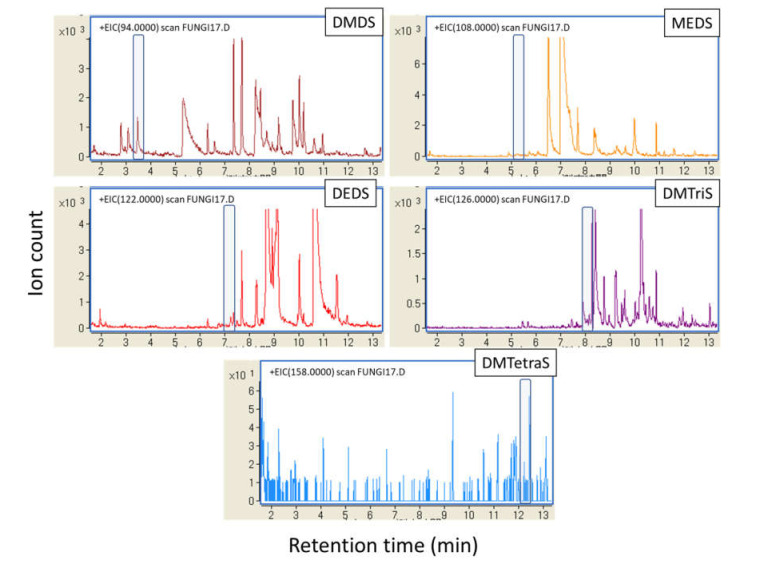
Extracted-ion chromatograms of VOCs from *A. fumigatus* IFM 51748. The gray-shaded area shows the putative retention time of each polysulfide.

Volatiles from *S. commune* strains cultured in slants with air-permeable silicone rubber plugs at 35 °C smelled like sulfur compounds. By GC/MS analysis using 49 *S. commune* strains cultured in securely sealed vials for 29 days, we detected five volatile sulfur compounds (VSCs) as major VOCs (Figure 1 and Table S2). Methyl ethyl disulfide (MEDS) was detected from every strain. DMDS was detected in all strains except for IFM 54722 strain. Diethyl disulfide (DEDS) and dimethyl trisulfide (DMTriS) were detected, respectively, from 34 and 40 strains. Dimethyl tetrasulfide (DMTetraS) was detected from 32 strains. DMTetraS-producing strains, except for IFM 58318, also produced DMDS and DMTriS. Strains not producing DEDS, except for IFM 58052 and IFM 55614, were confirmed as producing four other polysulfides. We analyzed the correlation of ion counts among detected sulfides. As shown in Figure 2, the ion counts of DMDS, DMTriS, and DMTetraS were correlated significantly with each other. The ion count of DEDS showed a weaker correlation with DMDS, DMTriS, or DMTetraS. We also examined GC/MS analysis for *Aspergillus* spp. presented in Table S1. The VSCs described above were not detected by GC/MS analysis (example data from the *A. fumigatus* IFM 51748 strain are shown in Figure 3). These data indicate that *S. commune*, but not *Aspergillus* spp., produces VSCs as major VOCs.

### 3.2. H_2_S Detection by Lead Acetate Paper

**Figure 4:**
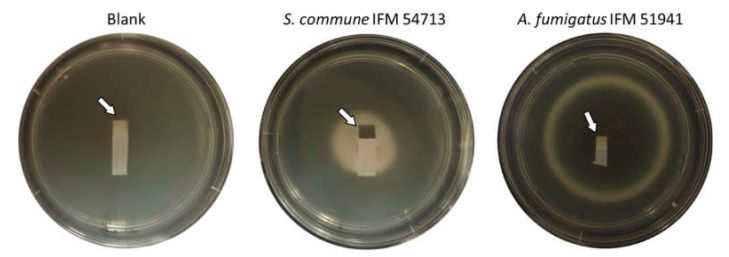
Detection of H_2_S using lead acetate paper. Lead acetate paper appearance exposed in a non-inoculated plate (**left**) and to VOCs from *S. commune* IFM 54713 (**middle**) and *A. fumigatus* IFM 51941 (**right**) for three days under 35 °C.

**Figure 5:**
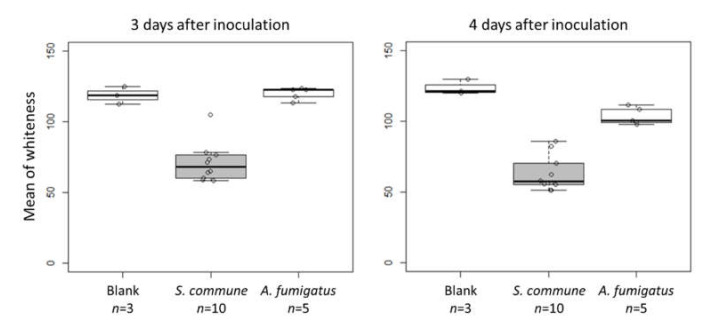
Detection of H_2_S using lead acetate paper. The whiteness of lead acetate.

We examined H_2_S detection using lead acetate paper. Three days after *S. commune* inoculation, a piece of lead acetate paper taped to the back of the agar plate lid turned black (Figure 4). The whiteness of lead acetate paper in plates with cultured *A. fumigatus* was comparable to that of blank plates (Figure 4 and Figure 5). Furthermore, DMDS or DMTriS on PDA did not turn lead acetate test paper black (data not shown). These data indicate that *S. commune* produces H_2_S as a major VSC, along with the polysulfides described above.

## 4. Discussion

Through this study, we determined VSCs including DMDS, DMTriS, DMTetraS, MEDS, DEDS, and H_2_S as the major VOCs of *S. commune*. Polysulfides are well-known odor VSCs [15,16]. Additionally, DMDS and DMTriS are distributed widely in cooked vegetables, beverages, and foods [17,18,19,20]. VSCs are produced by various microbes including bacteria (*Brevibacterium linens*) and yeasts (e.g., *Geotrichum candidum*, *Yarrowia lipolytica*, and *Candida* spp.) [21,22,23,24]. Among molds, truffles (*Tuber* spp.) are well-known producers of VSCs including DMDS and DMTriS [25]. Those VSCs are thought to be derived mainly from L-methionine or α-keto-γ-methylthiobutyric acid via methanethiol production [25]. Cystathionine γ-lyase and cystathionine γ-lyase produce methanethiol from L-methionine with lower efficiency. Cystathionine lyase homologs are found in *S. commune* [26]. Cystathionine γ-lyase was a highly expressed gene in fruiting bodies of *Tuber melanosporum*. Cystathionine γ-lyase is upregulated in fruiting bodies of *T. melanosporum* compared to free-living mycelia [27]. The reason why *S. commune* mycelia actively produced these VSCs remains unknown. Further research is expected to reveal the importance of these genes and the precise roles of VSCs.

As shown in Figure 2, dimethyl polysulfide production showed high correlation coefficients, suggesting that the methyl group is derived from methanethiol as described above. However, the correlation coefficients between DEDS and dimethyl polysulfides are very low (*R*^2^ is less than 0.02, not significant). The ethyl group in DEDS and MEDS might be derived from ethanethiol [28,29] because ethanol is a major VOC of *S. commune* [13] and because H_2_S can react with ethanol to give ethanethiol in vitro [28]. The difference between the methyl group and ethyl group supplies might be attributable to the low correlation coefficients of the ion peak area between DEDS and dimethyl polysulfides.

VSCs are found widely in nature as described above. Particularly, DMTetraS were found specifically as salival VSCs in patients with halitosis, but not in healthy individuals [30]. Additionally, DMTriS was detected frequently from samples of patients with halitosis, although it was also detected in healthy individuals [30]. Shirasu et al. reported DMTriS as a characteristic odor detected from fungating cancer wounds, although other sources such as associated bacteria contributed to the production [31]. In reports of both studies, the authors describe that polysulfides are candidates of biomarkers to diagnose specific diseases. The VSCs determined in *S. commune* culture might be candidates of biomarkers for the diagnosis of ABPM by *S. commune* from sputum, breath, and culture plates.

Furthermore, we observed that VOCs turned lead acetate paper black during *S. commune* culture. Lead acetate paper is commonly used to detect H_2_S sulfur odorants. Results show that *S. commune* produces H_2_S rapidly. Although the color of lead acetate paper was slightly changed (but comparable to control blanks) by the head space air of *A. fumigatus* under a four-day culture, the application also might be useful to determine *S. commune* as a causative agent of ABPM. Currently, we are trying to conduct research using clinical specimens.

Scott et al. demonstrated that volatile dimethyl sulfide and DMDS support *A. fumigatus* growth [32]. *Pseudomonas aeruginosa*, which is the major bacterial pathogen causing lung infections, is a producer of VSCs [32,33]. Similar to *P. aeruginosa*, VSCs from *S. commune* might support *A. fumigatus* growth in shared headspace. Both *S. commune* and *A. fumigatus* are causative agents of ABPM and fungal rhinosinusitis. Several coinfection cases have been reported [34,35,36]. Although the cases are rare, interspecies interaction might occur in the infection focus.

As described above, the production of polysulfides and H_2_S are known in some yeast species and bacterial species including *P. aeruginosa*. Therefore, these compounds might be less specific to identify *S. commune* using those compounds only. Additionally, some limitations are contained in this study. (1) The culture duration was quite long for the detection in this study. Our preliminary experiment showed that polysulfides were not detected in 7 days using three strains and detected from one of three strains at 14 days after inoculation. The examinations using seven additional strains showed that polysulfides were detected from two strains at 14 days after inoculation (Table S3). (2) While culturing with a crimp-capped vial, the O_2_ level is considered to be decreased. However, we did not monitor the O_2_ level in vials. In a gas-barrier pouch using H_2_S analysis, the O_2_ level was examined with an O_2_ monitor (OXY-2, Ichinen Jikco Co., Ltd., Tokyo, Japan). The data showed that O_2_ level in the pouch was gradually decreased but retained more than 15% for 9 days after inoculation of *S. commune* or more than 5% for 4 days after inoculation of *A. fumigatus*. The condition change might affect the VSC production during further cultivation. (3) In the colonization focus in the lung such as the mucus plug, *S. commune* is considered to grow aerobic on the surface and anaerobic in the plug. The in vivo condition is quite different from in vitro conditions. The VSCs production in patients with *S. commune* infection has never been examined. Further investigations related to growth condition and VSC production and the genetic and physiological mechanisms are expected to lead to the rapid and specific detection of VSCs and the identification of *S. commune* as the causative agent of ABPM and rhinosinusitis.

## 5. Conclusions

VSCs were identified as the major VOCs produced by *S. commune*, but not by *A. fumigatus*. H_2_S can also be detected from the headspace air of *S. commune* culture using lead acetate paper. These VSCs might be helpful as biomarkers to diagnose ABPM caused by *S. commune* and to identify the species from specimens.

## Data Availability

The data that support the findings of this study are available from the corresponding author upon reasonable request.

## Supplementary Materials

The following are available online at https://www.mdpi.com/article/10.3390/jof7060465/s1, Table S1: Fungal strains used in this study, Table S2: Abundance of polysulfides produced by *S. commune*, Table S3: Polysulfides detection at 14 days after culturing *S. commune* from the headspace air in each vial.
