# Supplementary material for: Identification of Volatile Sulfur Compounds Produced by Schizophyllum commune
Source: J Fungi (Basel). 2021 Jun 8;7(6):465. doi: 10.3390/jof7060465 (PMC8226890; doi:10.3390/jof7060465)
Supplement: Supplementary file 1 [file jof-07-00465-s001.zip › jof-1259381-supplementary/SupTables/SupTables.pdf]

Table S1. Fungal strains used in this study.

Species	Strain (IFM number)	Origin
Schizophyllum commune	54711	BALF
	54713	Sputum
	54716	Not recorded (clinically isolated)
	54717	BALF
	54718	Ear discharge
	54719	Not recorded (clinically isolated)
	54720	Mucus plug
	54721	Mucus plug
	54722	Middle nasal meatus
	55609	Nasal discharge
	55614	Sputum
	56967	BALF/mucus plug
	57114	Not recorded (clinically isolated)
	57137	Not recorded (clinically isolated)
	57149	BALF
	57155	Not recorded (clinically isolated)
	57162	Not recorded (clinically isolated)
	57165	Not recorded (clinically isolated)
	57462	Sputum
	58008	BALF
	58013	Mucus plug
	58014	Mucus plug
	58052	Mucus plug
	58101	BALF
	58102	BALF
	58318	Mucus plug
	58319	Mucus plug
	58323	Sputum
	58324	Sputum
	58326	BALF
	58333	BALF
	59086	Not recorded (clinically isolated)
	59343	BALF
	59347	Paranasal sinus
	59348	Not recorded (clinically isolated)
	59499	BALF
	59695	Lung
	59773	Unknown
	60198	Sputum
	60277	Peach (Ehime)
	60278	Environment (Tokyo)
	60279	Environment (Kyoto)

	60280	Environment (Fukushima)
	60281	Environment (Ibaraki)
	60282	Environment (Miyagi)
	60283	Environment (Okinawa)
	60284	Pear (*Pyrus pyrifolia*) (Saga)
	60285	Common grape vine (Ibaraki)
	60286	Apple (Aomori)
Aspergillus fumigatus	49824	BAL from ABPA patient
	49895	Lung
	51748	Sputum
	51941	Suction phlegm
	55369	Lung
	59777	Fungus ball in lung
Aspergillus niger	49437	Trachea
	49718	Sputum
	54606	Unknown
	57352	Unknown
	58160	Unknown
Aspergillus flavus	50915	Lung
	55547	Bronchoscope brushing
	56753	Lung
	57166	Unknown

BALF: bronchoalveolar lavage fluid.

Table S2. Abundance of polysulfides produced by *S. commune*.

Strain (IFM number)	Peak area				
	DMDS	MEDS	DEDS	DMTriS	DMTetraS
54711	132204	2044090	4253497	15030	ND
54713	38917	89506	35547	ND	ND
54716	100723	472114	264251	ND	ND
54717	594394	1653646	757664	70255	ND
54718	54534250	671145	ND	1130881	6785
54719	223127	934322	457274	65500	ND
54720	135820	741499	437060	33830	ND
54721	184596	2614023	4839444	96969	ND
54722	ND	926270	1280432	ND	ND
55609	229329	1363163	1055547	ND	ND
55614	958182	38026	ND	56284	ND
56967	71704718	4010629	75165	26856809	1129212
57114	6001930	12334243	2358861	2058957	29367
57137	288164	1104790	1044665	87271	2419
57149	106005	656610	631083	ND	ND
57155	12014586	15219097	1781985	8226981	477945
57162	20773366	274630	ND	2331450	37002
57165	48626	294028	197725	ND	ND
57462	2314596	20955182	17631918	223649	ND
58008	160342	1624691	1946920	71271	4545
58013	557254	6685949	9874627	929325	92924
58014	1014664	5866972	3880398	1035227	41603
58052	48976	100063	ND	ND	ND
58101	408156	6791557	13533691	252177	16470
58102	101774	1069331	1350632	ND	ND
58318	115472	796821	690827	ND	2774

58319	2779759	19614493	9239701	2341097	166183
58323	18871727	11463235	733077	10364417	354902
58324	6582306	2647209	176957	1924433	60058
58326	25050815	12730898	854293	3600431	161168
58333	353540	747600	291333	29316	ND
59086	6913229	115973	ND	1417057	13634
59343	4914089	16772609	5803659	5118986	255316
59347	91310981	50877226	5053172	37296338	3377840
59348	18616312	3079694	ND	7525293	385499
59499	345518	3727906	4520442	153176	6479
59695	7514307	26782879	12020516	3430772	94089
59773	137204	862349	595472	29147	ND
60198	26977214	256326	4594	1102210	10295
60277	15435370	204213	ND	2211150	34856
60278	13218015	170514	ND	930857	14155
60279	18174669	495377	ND	1236788	14297
60280	18398269	563981	ND	8587442	68323
60281	20711511	213136	ND	2458793	30589
60282	25476051	267485	ND	2156840	31336
60283	14039290	2192596	58140	1837609	20890
60284	25195522	132731	ND	1545653	14980
60285	2762203	68972	ND	359903	5161
60286	6276353	130290	ND	951193	8999

ND. not detected.

Table S3. Polysulfides detection at 14 days after culturing *S. commune* from the headspace air in each vial.

Strain (IFM number)	Peak area				
	DMDS	MEDS	DEDS	DMTriS	DMTetraS
54711					
54713					
54722					
55614					
57137					
58052					
58318	Detected	Detected	Detected	Detected	Detected
59347					
59695	Detected			Detected	Detected
60283	Detected			Detected	
60283					

Blank, not detected.
